# H1N1 challenge results in rapid recall of stem-specific immunity in HA stem nanoparticle–vaccinated newborn monkeys

**DOI:** 10.1172/jci.insight.194932

**Published:** 2025-11-10

**Authors:** Kali F. Crofts, Beth C. Holbrook, Courtney L. Page, Maya Sangesland, Masaru Kanekiyo, Martha Alexander-Miller

**Affiliations:** 1 Department of Microbiology and Immunology, Wake Forest University School of Medicine, Winston-Salem, North Carolina, USA.; 2 Vaccine Research Center, National Institute of Allergy and Infectious Diseases, NIH, Bethesda, Maryland, USA.

**Keywords:** Immunology, Infectious disease, Influenza, Vaccines

## Abstract

Primary exposure to influenza antigens during infancy shapes the humoral response to subsequent exposures. Development of a universal vaccine approach to protect newborns against influenza would represent a major step forward. In our previous study, we showed vaccination of newborn African green monkeys (AGMs) with an adjuvanted hemagglutinin (HA) stem nanoparticle induced robust IgG responses with broad recognition across HAs. Here, we examined the cellular responses in the lung-draining lymph node of these vaccinated newborn AGMs following challenge with a heterologous H1N1 virus. Our results show that vaccination is associated with early HA stem IgG^+^ B cell and antibody-secreting cell responses following infection, consistent with a rapidly recalled memory response. In addition, there was evidence of an increase in both HA stem– and head–specific plasma cells in vaccinated animals, suggesting a vaccine-engendered benefit for novel antibodies targeting HA epitopes. Finally, challenge was associated with preferential increases in antibodies that cross-react with H5 HA, suggesting improved protection against this divergent strain. Overall, these findings indicate that HA stem with AddaVax as adjuvant generates a stem-specific cross-reactive memory pool in newborn AGMs with the potential to be rapidly recalled upon infection.

## Abstract 2

Universal influenza vaccination in newborn monkeys generates memory responses that are quickly reactivated upon infection and help drive and shape the B cell response

## Graphical abstract

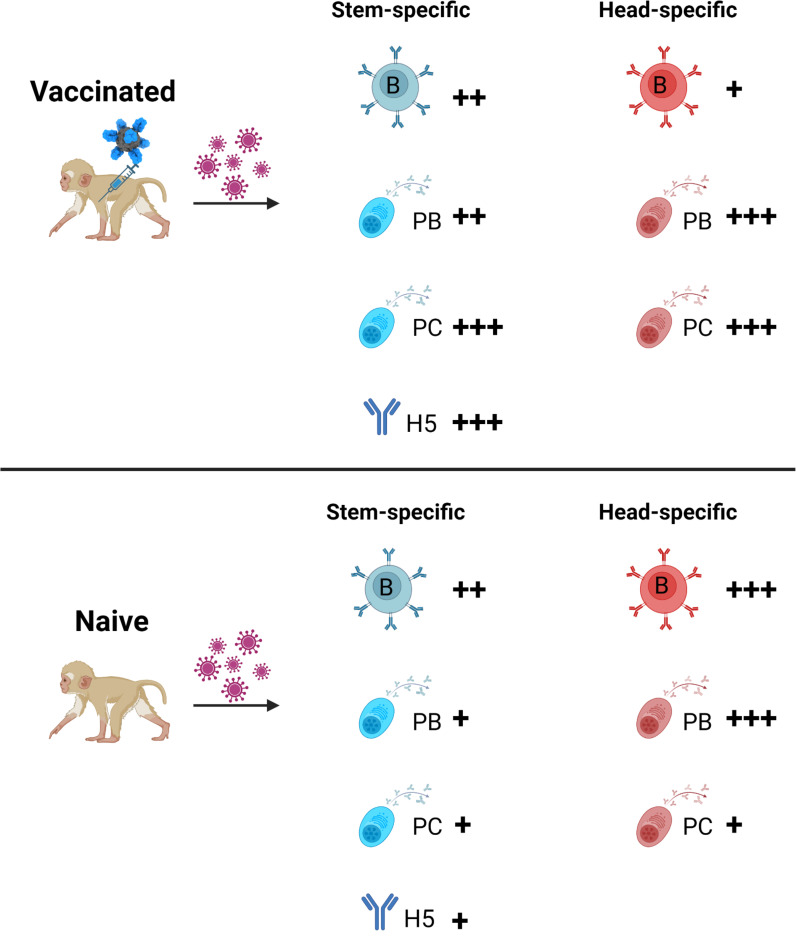

## Introduction

Influenza virus infections result in 290,000–650,000 deaths globally each year (1), with infants being particularly vulnerable to the development of severe disease. Annually, up to 374,000 individuals under 1 year of age are hospitalized with influenza virus infection, with 72% being less than 6 months old (2). This heightened susceptibility is due to both the changes associated with the immune system of newborns/young infants and the lack of an approved influenza vaccine for this age group. These factors highlight the urgent need for more effective vaccines that can provide protection in our youngest population.

There is compelling evidence that the first exposure to influenza virus shapes the humoral response to subsequent infection and vaccination (3–6), a phenomenon known as immune imprinting or original antigenic sin (7). Imprinting shapes subsequent immune responses to variant strains of influenza virus, resulting in a disproportionate skewing toward recall of memory B cells (MBCs) elicited by the priming event. This phenomenon can lead to poorer responses to a new influenza strain (4, 8). The majority (80%) of children are infected with influenza A virus (IAV) before the age of 3, suggesting that initial imprinting occurs during infancy and early childhood (6, 9). Supporting this, studies have shown that an individual’s birth year can strongly predict their antibody (Ab) response based on the strain of virus to which they were likely first exposed (10, 11). Individuals initially infected with a group 1 HA subtype of IAV develop different immunological memory and susceptibility to infection compared with those first exposed to a group 2 subtype. By way of example, during the 1918 H1N1 pandemic, young adults who had been infected with the H3N8 (group 2) strain during childhood experienced higher mortality to the 1918 H1N1 (group 1) virus than those initially exposed to a related H1N1 strain (12). Understanding how IAV vaccines shape this initial imprinting event during early life and subsequent protection against drifted influenza strains is crucial.

The newborn immune system is uniquely positioned to manage exposure to multiple environmental antigens as well as the establishment of the microbiome that occurs following birth. This is, in part, due to an altered immune system that exhibits diminished antigen-presenting cell (APC) function, lower production of proinflammatory cytokines, increased regulatory T cells, and a Th2-biased T cell response (13). Additionally, newborns have dampened germinal center (GC) responses, which reduces the generation of high-avidity Abs (14). These immune characteristics impact the generation of protective responses with current influenza vaccines, highlighting the challenges of effective vaccination in early life.

In addition to being poorly protective in young infants, current influenza vaccines offer limited protection against drifted or pandemic strains of influenza virus, as these vaccines mainly induce neutralizing Abs (nAbs) targeting the highly variable head region of hemagglutinin (HA) (15). This necessitates yearly updates, with variable effectiveness (10%–60%) based on the success of the inclusion of strains matching those in circulation (16). To address this, next-generation influenza vaccines are being developed that target less variable regions of the virus. One promising target for universal vaccination is the stem region of HA, which is relatively conserved due to its role in membrane fusion (17–19). A platform for eliciting responses to this region is a vaccine that displays a stabilized trimeric HA stem on the surface of a self-assembling ferritin (derived from *Helicobacter* *pylori*) nanoparticle (H1ssF) (20). In adult animal models, H1ssF delivered in the presence of squalene oil–in-water adjuvants elicits broadly neutralizing serological responses (20, 21). Additionally, a phase I clinical trial in adults has demonstrated that H1ssF increases the level of durable circulating nAb (22), as well as robust plasmablast (PB) and sustained MBC responses (23).

Recently, we assessed the effectiveness of the H1ssF vaccine administered to newborn African green monkeys (AGMs) in the presence or absence of the MF59-like adjuvant AddaVax (24). The nonhuman primate model is the most suitable preclinical newborn model available due to similarities with humans in the immune system as well as lung development and function (25, 26). This is coupled with a prolonged period of infancy compared with small animal models, which enables assessment of vaccine prime/boost and challenge responses during the newborn stage. In our previous analyses, we found prime/boost vaccination of newborn AGMs with H1ssF+AddaVax elicited high-titer, stem-specific Abs that were capable of broad recognition of group 1 HAs (24). However, despite generally high HA stem–specific Ab levels, the reduction in viral load in the lung on day 7 following challenge afforded by the vaccine-specific Ab response varied among animals (Supplemental Table 1; supplemental material available online with this article; https://doi.org/10.1172/jci.insight.194932DS1) (24). To understand differences in the vaccine-specific response that may have contributed to viral control, we assessed multiple measures of Ab quality, finding better control was associated with the presence of Abs with neutralizing and antibody-dependent cellular phagocytosis (ADCP) activity (24). Challenge of these vaccinated newborns afforded us the opportunity to probe the recall response present in the newborn AGM. The analysis of the cellular recall response in these animals is the focus of the studies presented here.

The nature of the recall response elicited in vaccinated newborns following challenge is an integral component of understanding vaccine efficacy. Here, we determined how the memory response and resulting imprinting established by the HA stem vaccine shaped the early B and T cell responses following challenge. To this end, we analyzed responses in the lung-draining tracheobronchial lymph nodes (TBLNs) after challenge with the pandemic H1N1 A/California/07/2009 (Ca09) virus. Newborns that received the H1ssF+AddaVax vaccine had increased levels of HA head– and stem–specific plasma cells (PCs) compared with nonvaccinated animals. Furthermore, newborns with vaccine responses that exhibited broader Ab effector function following vaccination and improved clearance had an increased stem-specific B cell recall response in the TBLNs. Additionally, IFN-γ–producing T follicular helper (Tfh) cell levels correlated with higher stem-specific PCs in H1ssF+AddaVax-vaccinated animals following challenge. This suggests Tfh recall responses play a crucial role in the differentiation into Ab-secreting cells (ASCs) early following infection in vaccinated newborns. Intriguingly, viral challenge modulated the pattern of reactivity across group 1 HA molecules, preferentially increasing Abs with H5 reactivity in the vaccinated newborns.

## Results

### Vaccination with H1ssF+AddaVax results in increased HA stem–specific cells within the IgG^+^ B cell response in the lung-draining TBLNs of Ca09-challenged infant AGMs.

**Figure 1:**
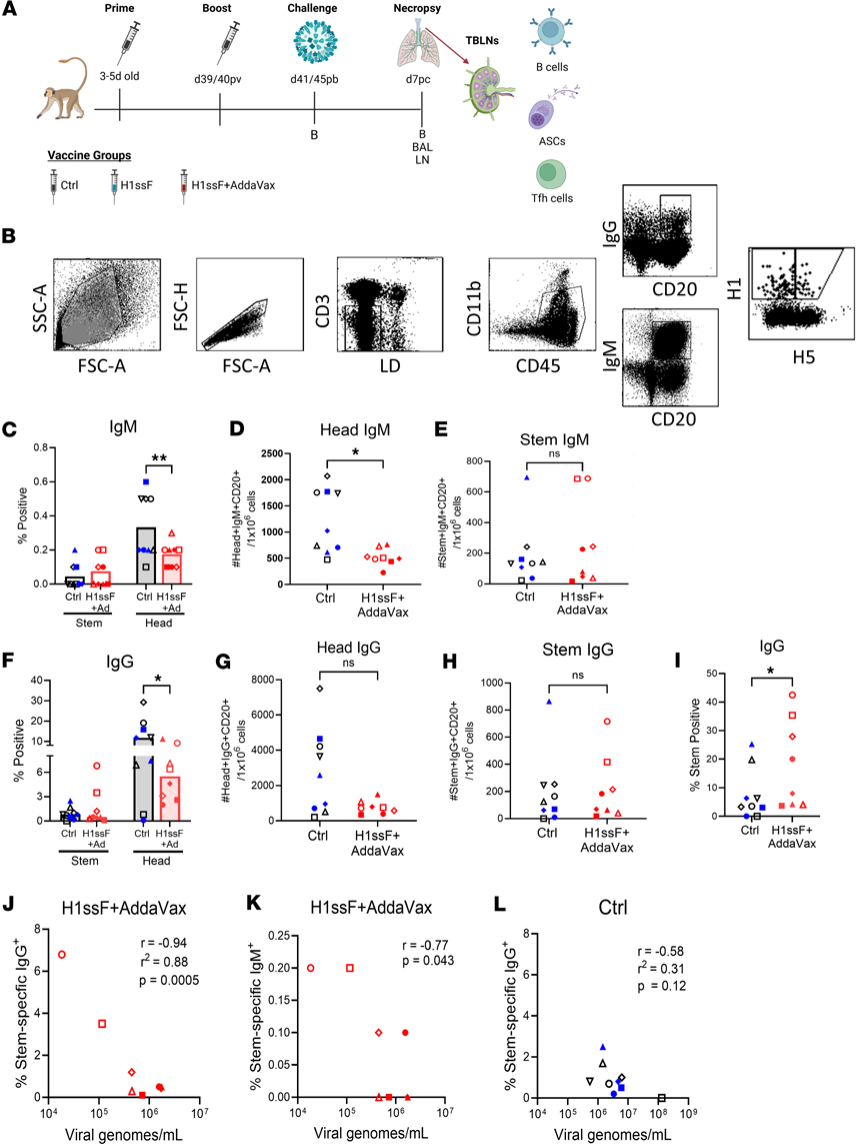
HA stem–specific cells are more highly represented within the IgG^+^ B cell response in the lung-draining TBLNs of Ca09-challenged infant AGMs vaccinated with H1ssF+AddaVax. (**A**) Newborn AGM vaccination and sampling schedule. (**B**) Representative gating strategy to identify CD20^+^IgM^+^ or IgG^+^ cells. HA head– (H1^+^H5^–^) and HA stem–specific (H1^+^H5^+^) B cells were identified using fluorescently labeled Ca09 HA (H1) and IN05 (H5) probes. (**C**) Percentage of HA head^+^ and stem^+^ within the CD20^+^IgM^+^ population. (**D**) Number of HA head^+^ and (**E**) stem^+^ cells within the CD20^+^IgM^+^ population. (**F**) Percentage of HA head^+^ and stem^+^ within the CD20^+^IgG^+^ population. (**G** and **H**) Number of (**G**) HA head^+^ and (**H**) stem^+^ cells within the CD20^+^IgG^+^ population. (**I**) Percentage of HA stem^+^ cells within the total HA^+^CD20^+^IgG^+^ population. (**J**–**L**) Correlation analysis comparing the viral genomes/mL in the BAL on day7 p.c. versus the percentage of (**J**) HA stem^+^CD20^+^IgG^+^ or IgM^+^ (**K**) cells in H1ssF+AddaVax animals or (**L**) versus HA stem^+^CD20^+^IgG^+^ in Ctrl animals. Based on the data distribution, a Pearson’s correlation analysis was used in **J** and **L** and a Spearman’s correlation analysis in **K**. Ctrl (PBS/Luc mRNA black symbols and H1ssF blue symbols, *n* = 9), H1ssF+AddaVax (red symbols, *n* = 8). Animals are assigned a distinct symbol that is used consistently throughout the data. Data in **C** and **F** represent the median. Statistical significance was determined using a 2-way ANOVA with an uncorrected Fisher’s LSD test (**C** and **F**) or a 2-tailed Mann-Whitney test (**D**, **E**, and **G**–**I**). Not significant (NS) *P* > 0.05, *P < 0.05, **P < 0.01. Viral genome data were previously published in Crofts et al. (24) and are shown here in alignment with author reuse policies of the publisher.

To establish an early-life immunity against HA stem, we immunized 3- to 5-day-old AGMs (equivalent to ~12- to 20-day-old humans) with H1ssF stem vaccine with or without AddaVax adjuvant in a prime/boost regimen (Figure 1A and Supplemental Table 2). As a nonvaccinated control, newborns received PBS or an mRNA lipid nanoparticle vaccine expressing luciferase (leveraged from an ongoing study). Vaccination with H1ssF+AddaVax induced high levels of HA stem–specific Ab in all newborns, with a subset producing Abs with neutralizing and ADCP activity (24). No difference in Ab level was detected by sex (Supplemental Figure 1).

Early following infection, MBCs enter newly formed GCs in the lung-draining LNs (27). Higher-affinity MBCs efficiently differentiate into ASCs (9). Naive B cells are also recruited into GCs, promoting diversification of the response to protect against drifted viral epitopes (28). We sought to understand how the H1ssF vaccine–induced response contributed to the number and specificity of B cells present in the lung-draining LNs following challenge with the heterologous H1N1 Ca09 virus in AGMs vaccinated as newborns. For these studies, TBLNs were isolated on day 7 following infection. For our analysis, nonvaccinated and H1ssF nonadjuvanted animals were combined and are referred to as the control (Ctrl) group moving forward, as we found no difference in Ab quantity or in viral load between the groups (Supplemental Figure 2).

As a first step, we probed the HA-specific (head and stem) B cell response in vaccinated and Ctrl animals following challenge. Antigen-specific IgM^+^ and IgG^+^ cells were evaluated within the CD20^+^CD3^–^CD45^+^CD11b^lo/–^ population. Head- and stem-specific B cells were distinguished using fluorescently labeled HA probes, Ca09 (H1) and A/Indonesia/05/2005 (IN05) (H5) (29). Costaining with these probes identified HA stem–specific B cells (H1^+^H5^+^), whereas HA head–specific B cells were positive only for the H1 probe (H1^+^H5^–^) (30) (Figure 1B and Supplemental Figure 3). Vaccinated newborns have prior exposure to HA stem, but are naive for HA head. Thus, on day 7 post challenge (p.c.), we anticipate the stem-specific response predominantly contains recalled MBCs, while the head-specific response reflects naive cells recruited by infection.

Analysis of IgM^+^ cells revealed a significant decrease in the percentage (Figure 1C) and number (Figure 1D) of HA head–binding cells in H1ssF+AddaVax animals compared with Ctrl animals, suggesting the presence of stem-specific memory cells may limit head-specific B cell recruitment into the response. No significant differences were observed in stem-specific IgM^+^ cells (Figure 1, C and E).

IgG is of high importance for influenza virus infection, as it is the predominant isotype responsible for protection in the lung (31). Similar to what we observed for IgM^+^ cells, evaluation of IgG^+^ cells revealed a decrease in the percentage of head-specific cells in the H1ssF+AddaVax group versus the Ctrl (Figure 1F). The number of head-specific cells was also lower, but this did not meet statistical significance, likely due to the heterogeneity in the Ctrl group (Figure 1G). Analysis of the number of stem-specific IgG^+^ cells revealed heterogeneity among the animals administered H1ssF+AddaVax, with a subset of the newborns showing an elevated percentage and number of stem-specific IgG^+^ cells (Figure 1, F and H).

We next sought to understand the relationship between the head- and stem-specific response following challenge in vaccinated and naive animals by evaluating the percentage of cells that bound HA stem within the total HA-specific B cell population. On average, stem-specific B cells were more highly represented in newborns that received H1ssF+AddaVax, comprising up to 43% of the IgG^+^ HA-specific cells (Figure 1I). Stem-specific IgG^+^ and IgM^+^ B cells were inversely correlated with viral load in the lungs — titers previously reported in Crofts et al. (24) — of vaccinated newborns following challenge (Figure 1, J and K). No significant correlation was found between viral load and the percentage of HA stem–specific IgG^+^ B cells in the Ctrl animals (Figure 1L). These data indicate that while H1ssF+AddaVax-vaccinated newborns generated a response to the head domain following challenge, it was dampened compared with Ctrl animals. Furthermore, within the HA-specific population, stem-specific B cells were significantly increased in representation in H1ssF+AddaVax compared with Ctrl animals. Finally, a higher early HA stem–specific B cell response in the TBLNs correlated with improved viral control following challenge.

### IFN-γ–producing HA stem–specific Tfh cells are associated with HA stem–pecific GC B cells in H1ssF+AddaVax newborns following challenge.

**Figure 2:**
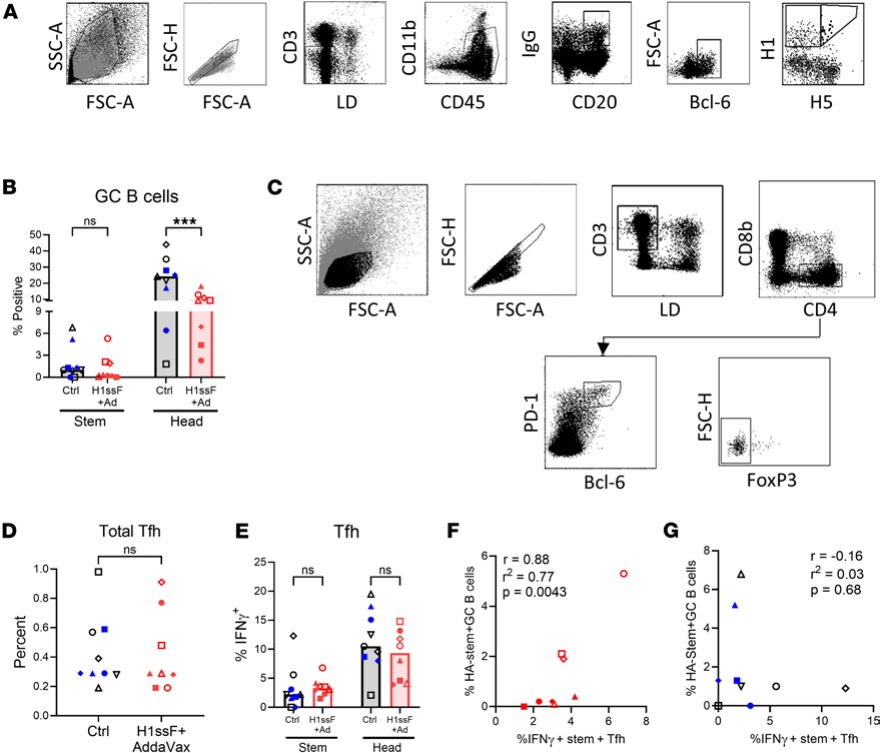
H1ssF+AddaVax promotes an IFN-γ–producing Tfh cell response that correlates with GC B cell responses on day 7 p.c. (**A**) Gating strategy to identify HA head– (H1^+^H5^–^) and HA stem–specific (H1^+^H5^+^) GC B cells (CD20^+^IgG^+^BCL-6^+^) in the TBLNs. LD (live/dead viability dye). (**B**) Percentage of HA head^+^ and stem^+^ cells within the GC population. (**C**) Gating strategy to identify Tfh cells (CD3^+^CD4^+^PD-1^hi^BCL-6^+^FoxP3^–^ cells). (**D**) Percentage of Tfh cells within the live CD3^+^ population. (**E**) Percentage of IFN-γ^+^ cells in the Tfh cell population following stimulation with Ca09 HA or NC99 stem peptide pools. (**F** and **G**) Pearson’s correlation was performed using the percentage of HA stem^+^ GC B cells and the percentage of IFN-γ^+^ Tfh cells in the TBLNs of H1ssF+AddaVax animals (**F**) or Ctrl animals (**G**) on day 7 p.c. Ctrl (PBS/Luc mRNA black symbols and H1ssF blue symbols, *n* = 9), H1ssF+AddaVax (red symbols, *n* = 8). Data in **B** and **E** represent the median. Statistical significance was determined using a 2-way ANOVA with an uncorrected Fisher’s LSD test (**B** and **E**) or a 2-tailed Mann-Whitney test (**D**). The first 4 panels in **A** are the same as in Figure 1B. Not significant (NS) *P* > 0.05, ***P < 0.001.

We next evaluated how vaccination with H1ssF+AddaVax impacted the generation of GC B cells on day 7 p.c. GC B cells were identified as IgG^+^CD20^+^BCL-6^+^; BCL-6 is the master transcriptional regulator of GC B cells and is essential for GC B cell development and maintenance (32). HA head^+^ or stem^+^ cells within the total GC population were evaluated (gating strategy shown in Figure 2A). Similar to what was observed in the analysis of the total IgG^+^CD20^+^ population, the percentage of head-specific IgG^+^ GC cells was significantly decreased in animals that had received the H1ssF+AddaVax vaccine compared with Ctrl animals (Figure 2B). Surprisingly, there was no difference in the percentage of HA stem–specific IgG^+^ GC cells (Figure 2B), nor the proportion of stem-specific cells that were of the GC phenotype (Supplemental Figure 4).

Tfh cells are important for the initiation and maintenance of the GC response (33). Tfh cells provide critical help to GC B cells through the secretion of cytokines and the expression of CD40L (33). The generation of Tfh responses is reduced in newborns with regard to both expansion and function, i.e., decreased cytokine production and CD40L expression (34–37). Tfh cells can acquire the capacity for production of distinct cytokines, e.g., IFN-γ versus IL-4 (38, 39). IFN-γ–producing Tfh cells have been reported to play a critical role in class switching and MBC accumulation in the lung following influenza virus infection (40). Given the importance of Tfh cells and IFN-γ, we evaluated total HA and stem-specific Tfh (CD3^+^CD4^+^PD-1^hi^BCL-6^+^FoxP3^–^) cells in TBLNs on day 7 p.c. (gating strategy shown in Figure 2C). IFN-γ production was assessed by stimulation of TBLN cells with either a Ca09-derived HA peptide pool or a stem peptide pool derived from H1N1 A/New Caledonia/20/1999 (NC99). At the group level, there was no significant difference in the total Tfh cells in the TBLNs (Figure 2D), the percentage of Ca09 HA–specific IFN-γ^+^ Tfh cells (Figure 2E), or the percentage of stem-specific IFN-γ^+^ Tfh cells (Figure 2E). Primary data are shown in Supplemental Figure 5, A and B. Additionally, there were no differences in the amount of IFN-γ produced on a per-cell level (Supplemental Figure 5, C and D).

Although there were no significant differences when group averages were compared, we noted the substantial heterogeneity within the Tfh and antigen-specific GC B cell populations. Thus, we explored whether there was a relationship between these populations that may provide mechanistic insights into the vaccine-mediated recall responses. We found that the number of stem-specific IFN-γ^+^ Tfh cells and stem-specific GC B cells in H1ssF+AddaVax-vaccinated animals were positively correlated (Figure 2F). In contrast, this correlation was not present in Ctrl infants (Figure 2G). Interrogation of HA stem–specific Tfh cells and head–specific GC B cells in H1ssF+AddaVax-vaccinated newborns revealed a correlation with a *P* value of 0.06, suggesting stem-specific Tfh cells may support the generation of head-specific GC B cells (Supplemental Figure 5E). No correlation was observed between HA head–specific Tfh cells and head–specific GC B cells in H1ssF+AddaVax-vaccinated newborns (Supplemental Figure 5F). Overall, these data support a model wherein newborns that produce stronger stem-specific Tfh cells following vaccination efficiently activate these cells following challenge that promotes early increases in HA stem–specific GC B cell responses.

### H1ssF+AddaVax prime/boost vaccination enhances HA stem–specific PB responses in the TBLNs following H1N1 challenge.

Following challenge with Ca09, H1ssF+AddaVax-vaccinated animals exhibited an Ab recall response characterized by boosted stem-specific Abs with neutralizing activity and an array of Fc effector activities (24). We expect this is the result of rapid differentiation of MBCs into Ab-secreting PBs following reexposure to antigen, while Ab responses to the head domain may be largely from extrafollicular responses at this early time point. Given this, we sought to understand how vaccination with H1ssF+AddaVax impacted the early generation of HA stem–specific versus head–specific PBs following infection.

**Figure 3:**
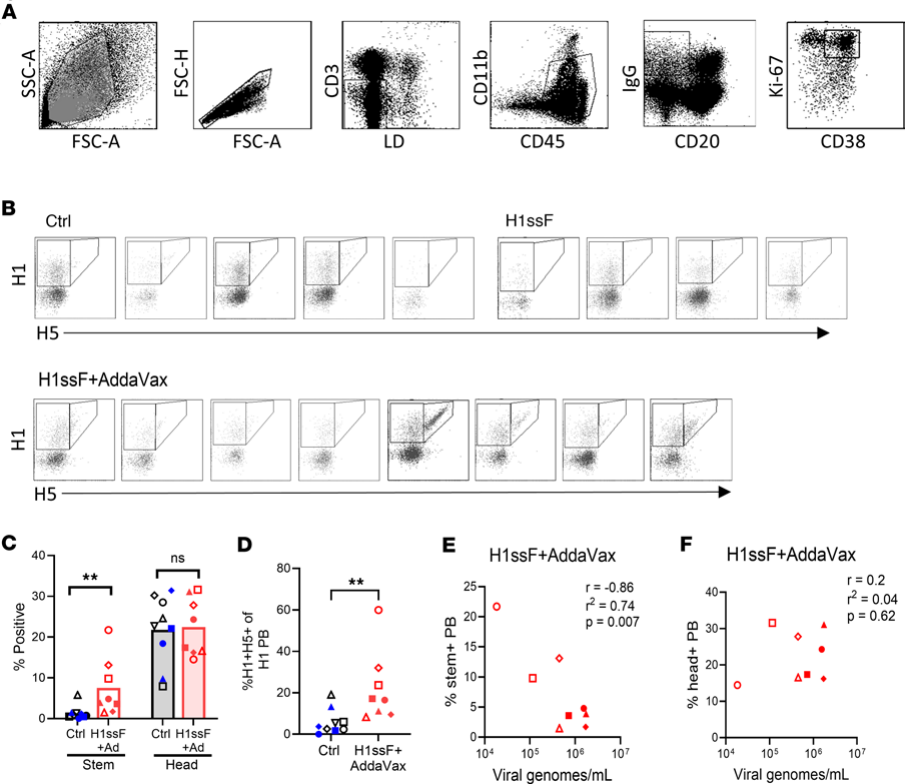
H1ssF+AddaVax promotes increases in the HA stem–specific PBs within the total H1 PB response in the TBLNs following challenge. (**A**) Gating strategy to identify PBs (CD20^–^IgG^+^CD38^+^Ki-67^+^) in the TBLNs. (**B**) HA head (H1^+^H5^–^) and HA stem (H1^+^H5^+^) cells within the PB population for individual animals. (**C**) Percentage of HA head^+^ and stem^+^ cells within the PB population. (**D**) Percentage of HA stem^+^ cells in the total H1^+^ PB population. (**E**) Pearson’s correlation analysis using the percentage of HA stem^+^ PB in the TBLNs on day 7 p.c. and viral genomes/mL in the BAL in H1ssF+AddaVax-vaccinated animals. (**F**) Pearson’s correlation analysis using the percentage of HA head^+^ PBs in the TBLNs on day 7 p.c. and viral genomes/mL in the BAL in H1ssF+AddaVax animals. Ctrl (PBS/Luc mRNA black symbols and H1ssF blue symbols, *n* = 9), H1ssF+AddaVax (red symbols, *n* = 8). Data in **C** represent the median. Statistical significance was determined using a 2-way ANOVA with an uncorrected Fisher’s LSD test (**C**) or a 2-tailed Mann-Whitney test (**D**). The first 5 panels in **A** are the same as in Figure 1B (alternative gating on panel 5). Not significant (NS) *P* > 0.05, **P < 0.01. Viral genome data were previously published in Crofts et al. (24) and are shown here in alignment with author reuse policies of the publisher.

PBs are marked by reduced CD20 expression, increased CD38, and high levels of proliferation (41). Here, PBs were identified as IgG^+^CD38^+^Ki-67^+^CD20^–^CD3^–^CD45^+^CD11b^lo/–^ cells (Figure 3, A and B). Cells were permeabilized prior to probe staining to allow detection of intracellular IgG (42), given the highly reduced expression of membrane-associated B cell receptor (BCR) in PBs (43). No significant differences were observed in the percentage (Figure 3C) or number (Supplemental Figure 6A) of H1^+^H5^–^ head-specific PBs with vaccination. In contrast, vaccination resulted in a higher percentage (Figure 3C) and number (*P* = 0.07) (Supplemental Figure 6B) of stem-specific PBs. Analysis of the stem-specific response within the total H1 PB population showed a higher proportion of cells reactive to the stem domain in the H1ssF+AddaVax-vaccinated animals compared with Ctrl animals (Figure 3D). Together, these data suggest newborn vaccination with H1ssF+AddaVax promotes an increased stem-specific PB response early following challenge, while also allowing for emergence of head-specific ASCs.

We further asked whether the day 7 p.c. PB response correlated with viral clearance. Intriguingly, HA stem–specific (Figure 3E), but not head–specific (Figure 3F), PBs correlated with reduced virus in the lung of H1ssF+AddaVax infants. These data suggest that a robust stem-specific PB response is recalled from the H1ssF+AddaVax-induced memory pool, which is associated with decreased viral load in the lung following challenge.

### H1ssF+AddaVax prime/boost vaccination of newborns results in increased stem- and head-specific PCs in the TBLNs following H1N1 challenge.

**Figure 4:**
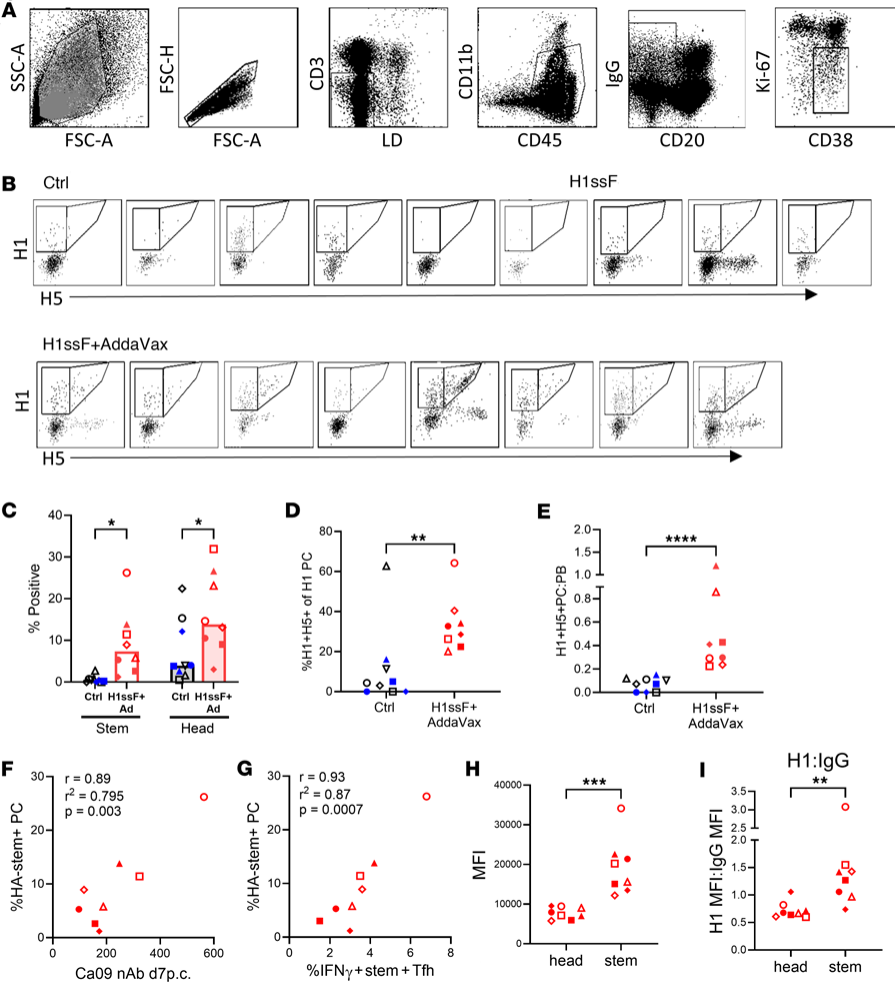
H1ssF+AddaVax promotes increases in HA stem– and head–specific PCs in the lung-draining LNs following challenge. (**A**) Gating strategy to identify PCs (CD20^–^IgG^+^CD38^+^Ki-67^–^) in the TBLNs on day 7 p.c. (**B**) Flow cytometric analysis of HA head (H1^+^H5^–^) and HA stem (H1^+^H5^+^) cells within the PC population for each animal. (**C**) Percentage of HA head^+^ and HA stem^+^ cells within the PC population. (**D**) Percentage of HA stem^+^ cells within the total H1^+^ PC population. (**E**) Ratio of stem PCs/10^6^ cells to stem PBs/10^6^ cells. (**F**) Pearson’s correlation analysis using the percentage of HA stem^+^ PCs in the TBLNs and nAb in the plasma on day 7 p.c. in H1ssF+AddaVax animals. The nAb titers were previously reported in Crofts et al. (24). (**G**) Pearson’s correlation analysis of the percentage of HA stem^+^ PCs and percentage of IFN-γ^+^stem^+^ Tfh cells in the TBLNs on day 7 p.c. in H1ssF+AddaVax animals. (**H**) The MFI of H1 probe binding in stem- and head-specific PCs in the TBLNs of H1ssF+AddaVax animals. (**I**) Ratio of H1 probe binding to IgG in PCs. Ctrl (PBS/Luc mRNA black symbols and H1ssF blue symbols, *n* = 9), H1ssF+AddaVax (red symbols, *n* = 8). Data in **C** represent the median. Statistical significance was determined using a 2-way ANOVA with an uncorrected Fisher’s LSD test (**C**) or a 2-tailed Mann-Whitney test (**D**, **E**, **H**, and **I**). The first 5 panels in **A** are the same as in Figure 1B. *P < 0.05, **P < 0.01, ***P ≤ 0.0005, ****P ≤ 0.0001. nAb data were previously published in Crofts et al. (24) and are shown here in alignment with author reuse policies of the publisher.

We next evaluated the impact of the vaccine-induced response on the generation of HA head– and stem–specific PCs, defined as CD38^+^Ki^–^67^lo/–^CD20^–^IgG^+^ (Figure 4, A and B). We found a significant increase in both HA stem– and head–specific PCs in H1ssF+AddaVax animals compared with the Ctrl group at this early time point (Figure 4C and Supplemental Figure 6, C and D); however, the increase in number was more robust in the stem-specific PCs, a 16-fold increase versus 2.2-fold in the head-specific PC population. As a result, HA stem–specific PCs comprised an increased percentage of the HA-specific response in H1ssF+AddaVax-vaccinated animals compared with Ctrl animals (Figure 4D). Furthermore, H1ssF+AddaVax animals had increased stem-specific PC/PB ratios compared with Ctrl animals, consistent with enhanced differentiation into PCs following challenge (Figure 4E). Overall, these data show prior vaccination of newborns with H1ssF+AddaVax results in a significantly enhanced generation of both HA stem– and head–specific PCs in the lung-draining TBLNs after challenge. We observed a strong correlation between the percentage of HA stem–specific PCs and both the level of nAb (Figure 4F) and the IFN-γ^+^ Tfh cells (Figure 4G) in H1ssF+AddaVax-vaccinated newborns. These correlations were not present in Ctrl animals (Supplemental Figure 6, E and F).

The finding of increased head-specific PCs in H1ssF+AddaVax-vaccinated newborns was somewhat unexpected given the decreased head-specific IgG^+^ B cells and the lack of correlation between Tfh and head-specific GC B cells. Given the naive status of head-specific B cells in these animals prior to challenge, we reasoned that head-specific B cells may be the result of extrafollicular responses, while stem-specific cells may be dominated by recalled MBCs. If this were the case, we reasoned stem-specific cells would have undergone affinity maturation in the H1ssF+AddaVax-vaccinated animals. If so, we would expect higher binding to the H1 probe in stem-specific compared with head-specific PBs. To test this possibility, we analyzed the level of H1 probe binding in the 2 populations. Stem-specific PCs had significantly higher levels (2.5-fold) of H1 binding compared with head-specific cells (Figure 4H). Differences in IgG level could not explain this result, as stem-specific probe binding remained significantly higher when normalized to IgG (Figure 4I).

### H1N1 Ca09 challenge increases the relative representation of H5-binding Abs in H1ssF+AddaVax-vaccinated newborns.

**Figure 5:**
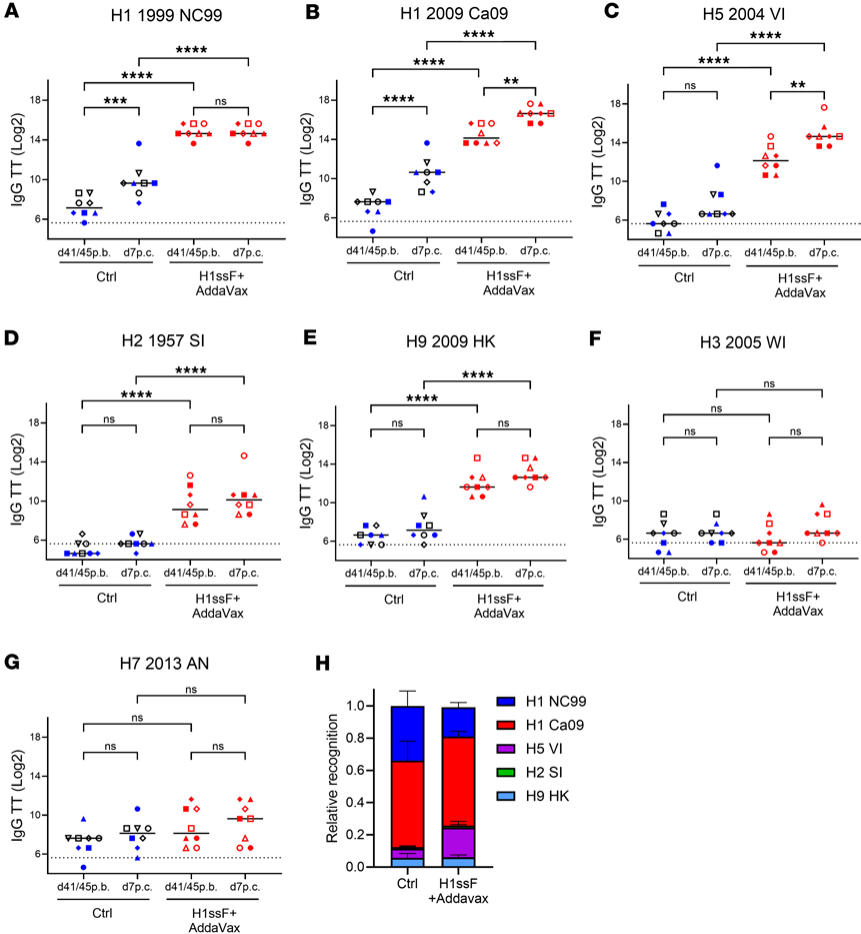
H1N1 challenge of H1ssF+AddaVax-vaccinated infant AGMs preferentially promotes increases in H5-binding IgG on day 7 p.c. Group 1 and group 2 HA–specific IgG binding was measured in the plasma on day 41/day 45 p.b. and day 7 p.c. Group 1 HA subtypes include A/New Caledonia/20/1999 (H1 1999 NC99) (**A**), A/California/07/2009 (H1 2009 Ca09) (**B**), A/Vietnam/1204/2004 (H5 2004 VI) (**C**), A/Singapore/1/1957 (H2 1957 SI) (**D**), and A/Hong Kong/33982/2009 (H9 2009 HK) (**E**). Group 2 HA subtypes include A/Wisconsin/67/2005 (H3 2005 WI) (**F**) and A/Anhui/1/2013 (H7 2013 AN) (**G**). The dotted line represents the limit of detection for the assay. (**H**) The average representation of each HA on day 7 p.c. for each animal. Threshold titer (TT) was defined as the highest dilution resulting in an OD_450_ greater than 3 times the assay background. Ctrl (PBS/Luc mRNA black symbols and H1ssF blue symbols, *n* = 9), H1ssF+AddaVax (red symbols, *n* = 8). Data represent the median. Statistical significance was determined using a 1-way ANOVA with Tukey’s post hoc analysis. Not significant (NS) *P* > 0.05, **P < 0.01, ***P < 0.001, ****P ≤ 0.0001.

At present, we have a limited understanding of how infection modulates the stem-specific Ab response generated following imprinting through HA stem vaccination. To gain insights into this question, we interrogated the HA-binding profiles of Abs present on day 7 p.c. This time point reflects recruitment of both naive B cells (as demonstrated by HA head–reactive ASCs in vaccinated newborns) and HA stem–reactive B cells, which are most likely dominated by vaccine-elicited memory B cells. Vaccination with H1ssF+AddaVax in newborns resulted in IgG that was broadly reactive across group 1 HA molecules on day 41/45 post boost (p.b.) (24). These previously reported data are shown here for comparative purposes (Figure 5). Challenge resulted in selective increases in H1 Ca09– and H5-binding Abs (Figure 5, B and C). The increase in Ca09 titers (Figure 5B) likely reflects Ab responses targeting the head region as well as stem. We propose the significant increase in H5 IgG (Figure 5C) is the result of the preferential recruitment of stem-specific MBCs with higher affinity for H5 following Ca09 infection.

While the response in the Ctrl animals was modest on day 7 following infection, we sought to understand the breadth of reactivity generated against the group 1 HA molecules at this time in the naive setting. The data in Figure 5H represent the titer of the Ab response to each HA type in relation to the sum of the responses for both H1ssF+AddaVax-vaccinated and Ctrl animals. Vaccinated animals showed increased H5 reactivity along with a decreased response to NC99. Thus, the reactivity pattern across HA molecules of Abs generated early following infection is modified in newborns by prior vaccination with an H1 NC99 stem construct, exhibiting skewing toward H5 reactivity.

## Discussion

An understanding of how vaccination in naive newborns impacts immune imprinting is foundational for the development of an effective universal influenza vaccine for young infants. Here, we explored the early cellular and Ab responses generated following H1N1 Ca09 challenge of H1ssF+AddaVax-vaccinated newborn AGMs. To our knowledge, this is the first study to evaluate the cellular immune response following challenge in a young infant model administered a universal HA stem vaccine. We found vaccination resulted in an increase in HA stem–specific IgG^+^ PCs and PBs within the HA-specific B cell pool in the lung-draining TBLNs on day 7 p.c. This was accompanied by a general decrease in head-specific responses. Furthermore, the level of IFN-γ–producing stem-specific Tfh cells was positively correlated with the magnitude of the HA stem–specific GC B cells following challenge.

Interestingly, we observed a greater number of both stem- and head-specific PCs in vaccinated animals following challenge. The recall responses evaluated here were imprinted by vaccination with an HA stem–bearing nanoparticle. Thus, the HA-specific immune response was focused solely on the stem region. This is highly advantageous given the well-established subdominant nature of this response in the context of the full HA molecule. Vaccination with the seasonal inactivated vaccine induces a highly limited Ab response to the stem region compared with that directed to the head domain (44). At present, it is not clear whether or how the repertoire of responding stem-specific B cells may differ when elicited in the presence versus the absence of the immunodominant HA head. However, the recruitment of a distinct population of B cells in the memory pool with a stem-based vaccine that differed in avidity or specificity could potentially impact the recall response. In addition to the cellular recall response, we evaluated how challenge with Ca09 modified the H1ssF+AddaVax-induced Ab pool, finding selective increases in Abs that recognize H5. Together, our findings show that newborn vaccination with H1ssF+AddaVax results in an HA stem–specific memory response that is efficiently recalled and tuned following H1N1 challenge. Furthermore, the memory stem-specific response appears to facilitate both an early HA stem– and head–specific ASC response.

As in humans, we observed heterogeneity in viral clearance (24) and the HA stem–specific recall response in our newborn AGMs. As a first step in exploring these differences, we probed maternal age, maternal weight, maternal waist circumference, and social rank as potential modulating factors. None of these correlated with the magnitude of the vaccine or recall response in the newborns. The AGM model employed here is genetically restricted, but not inbred. In fact, 7 of the 8 infants vaccinated with H1ssF+AddaVax were sired by males introduced into the colony in 2018 to maintain diversity (see pedigree and kinship coefficients in Supplemental Figure 7). We propose that the genetic diversity in the newborns may contribute to the variability in the vaccine response and the recall response.

Promoting MBC responses is paramount to successful vaccination, as these cells can rapidly differentiate into ASCs upon reexposure to antigen (45). This is, in part, the result of both higher affinity of MBC BCRs for antigen and a lower activation threshold compared with naive B cells (46, 47). In human adults, who have had multiple IAV exposures through both infection and vaccination, H1ssF has been shown to increase circulating MBCs and PBs (23). In our studies, TBLNs from naive newborn AGMs that were vaccinated and boosted with H1ssF+AddaVax were analyzed on day 7 following virus challenge. At this time, vaccine-elicited MBCs would likely have acquired 1 of 2 fates: differentiation into ASCs or entry into an emerging GC response. Our data showed enrichment of HA stem–specific PBs relative to the total H1-specific PB population along with enhanced HA stem–specific PB and PC responses in the H1ssF+AddaVax-vaccinated infants. These findings suggest efficient differentiation of vaccine-elicited B cells into ASCs following challenge. Interestingly, we also observed an increase in the HA head–specific PC response, which was unexpected given the naive status of the animals and this early time point. One possibility could be that head-binding PCs are derived from extrafollicular responses that are supported by stem-specific non-GC Tfh memory CD4^+^ T cells (48) generated by H1ssF+AddaVax vaccination.

Our current understanding of imprinting by influenza virus infection or vaccination in newborns is highly limited. The development of a universal vaccine raises an important question of how imprinting on a conserved region, such as the HA stem, impacts future responses to infection and/or vaccination. A robust stem-specific response would offer substantial benefit through protection against pandemic strains and provision of multi-season immunity. Our studies show that priming infants with H1ssF+AddaVax results in higher stem-specific cellular responses following challenge with heterologous H1N1, as evidenced by increased stem-specific PB and PC responses compared with Ctrl infants. Intriguingly, we observed lower HA head–specific GC B cells in the vaccinated versus Ctrl infants. These findings are consistent with a model wherein priming with the stem region promotes continued higher representation of stem-specific responses within the GC repertoire in the face of infection, although admittedly we have only evaluated the early response. A study by Hensley and colleagues examined the impact of heterosubtypic infection on the stem-specific Ab response generated by infection of mice (49). Surprisingly, although Abs against the priming HA stem were boosted, they exhibited poor binding to the heterosubtypic HA stem. In our study, we found infection was associated with increased recognition of the heterosubtypic H5 molecule. The apparent broadening of the response observed here may reflect differences in the Ab pool elicited by the H1ssF vaccine or vaccination in general, the impact of adjuvant, or inherent properties of the newborn immune system. In studies of adult rhesus macaques, the Ab repertoire generated by infection versus HA vaccination was found to differ with regard to epitope specificity (50). Whereas infection resulted in a memory B cell response with neutralizing activity but little forward breadth, the response following vaccination, while having limited neutralizing activity, exhibited substantially greater breadth of binding (50). In addition, regardless of the priming event, boosting with the homologous recombinant HA protein resulted in increased breadth of recognition. Further studies are needed to fully understand how future encounters with influenza virus and vaccines modulate the long-term response imprinted in newborns by an HA stem vaccine.

We previously established that prime/boost vaccination with H1ssF+AddaVax in newborns induced a polyclonal IgG response with broad recognition across group 1 HA subtypes (24). As noted above, following challenge, newborns exhibited a preferential increase in Abs capable of recognizing H5. Following vaccination, recognition of H5 was approximately 6-fold lower than for NC99 H1 (24). Remarkably, by day 7 p.c., binding to H1 and H5 was similar. Recognition of H5 by Abs induced by full-length H1 HA has been reported (51, 52). Not surprisingly, these Abs were shown to primarily recognize epitopes in the stem region. Furthermore, the H1-elicited Abs provided protection against H5 challenge in adult mouse and ferret models (51, 52). Our findings suggest Ca09 infection promotes preferential recall of NC99 stem–elicited MBCs that have the potential to bind to H5. In addition, they lead to the appealing hypothesis that H1ssF vaccination recruits B cells bearing receptors with a propensity for H5 recognition that are further boosted with heterologous H1N1 virus infection.

Our study has limitations. The data presented here reflect a single time point, day 7 p.c., shortly following H1N1 challenge, leaving the temporal dynamics of the cellular immune response in TBLNs unexplored. Thus, while we can draw conclusions about how prime/boost vaccination with the H1ssF+AddaVax vaccine influences the early stem-specific B cell and Ab response, it is uncertain how these responses might evolve over time after the GC has fully matured, an area that warrants further investigation.

Overall, our studies demonstrate that vaccination with H1ssF+AddaVax enhances the HA stem–specific Abs present following H1N1 IAV challenge. These findings extend and complement our previous work showing that H1ssF+AddaVax can generate Ab responses that promote viral clearance (24). Here, we show that vaccination results in an increased HA stem–specific GC response that is associated with IFN-γ–producing stem-specific Tfh cells early following challenge. Furthermore, vaccination appears to elicit Tfh cells that support stem-specific B cell and GC B cell responses following challenge. In addition, there is evidence of an increase in both HA stem– and head–specific PCs in vaccinated animals, suggesting a vaccine engendered benefit for novel Ab-targeted HA epitopes. Finally, stem-specific B cells remain more highly represented in vaccinated animals following challenge. With regard to Ab reactivity, the early recall response in vaccinated animals exhibits a preferential increase in Abs reactive to H5. Boosting these broadly reactive Abs established by stem immunization through H1N1 exposure may offer enhanced protection against H5 strains with pandemic potential. Understanding how stem-based vaccines shape the infant immune response to influenza virus and the potential for broadening the breadth of protection against IAV infection may help to overcome the challenge of generating immunity against continually evolving seasonal and pandemic IAV strains.

## Methods

### Sex as a biological variable.

Sex was considered as a biological variable, with equally matched numbers of male and female AGMs. No differences by sex was observed.

### Study design.

AGMs were housed, bred, immunized, and sampled at the Vervet Research Colony at the Wake Forest University School of Medicine. Healthy newborn AGMs weighing over 300 g were immunized between 3 and 5 days after birth, which corresponds to approximately 12 to 20 days of human infancy. A total of 17 newborn AGMs were randomly assigned to the following experimental groups: PBS or luciferase mRNA-LNP (non–HA-vaccinated controls) (Ctrl) (*n* = 5), H1ssF (*n* = 4), and H1ssF+AddaVax (*n* = 8). The H1ssF vaccine is comprised of the stabilized stem domain of HA from NC99 displayed on the exterior of a self-assembled *H*. *pylori* ferritin nanoparticle (20). For construction of the stabilized stem, the receptor-binding domain (HA1 residues 51–277, H3 numbering) was replaced with a glycine-serine-glycine (GSG) linker, and HA2 residues 58–93 were connected using a loop comprised of glycine and serine residues (GSGGSG). Mutations (K51M and E103L) were introduced to stabilize the inner core of the HA stem. The stabilized stem subunit was genetically fused to the N-terminus of *H*. *pylori* ferritin subunit (residues 5–167) with a serine-glycine-glycine (SGG) linker so that 8 copies of the HA stem trimers are formed at the 3-fold axes on a self-assembled 24-mer ferritin nanoparticle. H1ssF was produced in mammalian cells (Expi293) by transient transfection and purified from culture supernatant by a lectin chromatography followed by a size exclusion chromatography, as described previously (20). H1ssF was made in the laboratory of Masaru Kanekiyo at the NIH Vaccine Research Center (VRC) where the vaccine was developed. No significant differences were observed between the PBS/luciferase mRNA-LNP control group and the H1ssF nonadjuvanted group, so they were combined into a single control group (Ctrl) for further analyses. Animals were balanced by sex across groups to ensure even male and female distribution. Newborns were immunized intramuscularly with 20 μg of H1ssF, with or without AddaVax (125 μL; InvivoGen), into the deltoid muscle of both left and right arms (totaling 40 μg of H1ssF per dose). A booster dose was given on day 39/day 40 post vaccination (p.v.). Plasma samples were collected at several time points: day 1 p.v., day 10 p.v., day 39/day 40 p.v., day 10/day 11 p.b., day 41/day 45 p.b., and day 7 p.c. On day 41/day 45 p.b., all animals were challenged with 1 × 10^7^ TCID_50_ (0.75 mL) of Ca09 (BEI Resources) (0.5 mL given by intratracheal installation, and 0.125 mL in each nostril). Bronchoalveolar lavage (BAL) samples were collected on day 7 p.c. All animals were euthanized on day 7 p.c., and lung-draining TBLNs were collected at necropsy for further analyses. TBLNs were processed into single-cell suspensions and stored in liquid nitrogen.

### Flow cytometry for the detection of H1^+^ and H5^+^ B cell and ASC responses.

TBLNs were thawed and 1 × 10^6^ cells added to 96-well round-bottom plates for staining. Cells were pelleted and stained with the Zombie Aqua fixable viability dye (BioLegend) to exclude dead cells. Subsequently, 2.5 μg of Fc Block (BD Biosciences, 564220) was added to prevent nonspecific binding. To identify influenza HA head– and HA stem–specific B cells, the cells were stained with fluorescently labeled HA probes Ca09 (H1) and IN05 (H5), both from the NIH VRC. HA stem–specific B cells were identified as double-positive (H1^+^H5^+^), while HA head–specific B cells stained positive only for the H1 probe (H1^+^H5^–^). Brilliant Stain Buffer (BD Biosciences, 563794) was used to optimize staining.

The surface Ab panel included BUV395 anti-IgM (G20-127) (BD Biosciences, 563903), BV510 anti-IgG (G18-145) (BD Biosciences, 563247), FITC anti-CD38 (AT-1) (STEMCELL Technologies, 100-1578), PeVIO770 anti-CD3 (10D12) (Miltenyi Biotec, 130-116-628), AF700 anti-CD20 (2H7) (BD Biosciences, 560631), V450 anti-CD45 (D058-1283) (BD Biosciences, 561291), BV786 anti-CD11b (M1/70) (BD Biosciences, 740861), PE-conjugated H1 probe (VRC, NIH), and APC-conjugated H5 probe (VRC, NIH).

Following surface staining, cells were permeabilized for 1 hour at room temperature in the dark using the FoxP3/Transcription Factor Buffer Set (eBioscience, 00-5523). Intracellular staining was then performed to assess Ki-67 and BCL-6 expression and intracellular binding of HA probes to BCRs/Abs (42). Abs used for intracellular staining included BUV395 anti-IgM (G20-127) (BD Biosciences, 563903), BV510 anti-IgG (G18-145) (BD Biosciences, 563247), BUV737 anti–Ki-67 (B56) (BD Biosciences, 567130), PerCpCy5.5 anti–BCL-6 (K112-91) (BD Biosciences, 562198), PE-conjugated H1 probe (VRC, NIH), and APC-conjugated H5 probe (VRC, NIH). Samples were acquired on an BD LSR Fortessa X-20 (BD Biosciences) and analyzed with BD FACSDiva software (BD Biosciences).

### Flow cytometry for the detection of Ca09 HA– and NC99 stem–specific T cell responses.

TBLNs were thawed and 1 × 10^6^ cells added to 96-well flat-bottom plates for stimulation in RPMI-1640 medium supplemented with 2 mM L-glutamine, 0.1 mM sodium pyruvate, 1× nonessential amino acids, 100 U/mL penicillin, 100 μg/mL streptomycin, 10 mM HEPES (Gibco), 0.05 mM 2-mercaptoethanol (Sigma-Aldrich), and 10% fetal bovine serum (Atlanta Biologics). Cells were then stimulated with peptide pools. HA stem peptide pools were derived from the influenza A/New Caledonia/20/1999 (H1N1) HA protein (BEI Resources, NR-2602). Pools included peptides 2–10 (D18–L58) and 50–90 (A292–I529) to target the stem region of HA (53). Each peptide was 16–17 amino acids in length, overlapping by 11–12 amino acids. Peptides were reconstituted in DMSO to achieve a final well concentration of 0.2% DMSO (Sigma-Aldrich, D2650). For stimulation with Ca09 HA, PepMix from influenza A (HA/Cal/H1N1) (JPT Peptide Technologies, 56080) was used, also reconstituted in DMSO (final concentration of 0.2%). The final concentration of each peptide was 0.5 μg/mL for NC99 HA and 1 μg/mL for Ca09 HA PepMix. Intracellular cytokine production was assessed after 14 hours of stimulation with the presence of brefeldin A (1:1,000; GolgiPlug, BD Biosciences) and monensin (1:1,500; GolgiStop, BD Biosciences) from 3–14 hours.

Following stimulation, cells were transferred to 96-well round-bottom plates. Cells were pelleted and stained with Zombie Aqua fixable viability dye (BioLegend) to exclude dead cells. Subsequently, 2.5 μg of Fc Block (BD Biosciences, 564220) was added to prevent nonspecific binding. Brilliant Stain Buffer (BD Biosciences, 563794) was used to optimize staining. The surface Ab panel included BV650 anti-CD4 (L200) (BD Biosciences, 564220), PE anti-CD8b (2ST8.5H7) (Beckman Coulter, IM2217U), PE/Dazzle 594 anti–PD-1 (EH12.2H7) (BioLegend, 329940), PeVIO770 anti-CD3 (10D12) (Miltenyi Biotec, 130-116-628), and AF700 anti-CD20 (2H7) (BD Biosciences, 560631). Abs used for intracellular staining included BUV395 anti–IFN-γ (B27) (BD Biosciences, 563563), BUV737 anti–Ki-67 (B56) (BD Biosciences, 567130), AF488 anti-FoxP3 (206D) (BioLegend, 320112), and AF647 anti–BCL-6 (K112-91) (BD Biosciences, 561525). Samples were acquired on a BD LSR Fortessa X-20 and analyzed with BD FACSDiva software.

### ELISA for the detection of broadly reactive HA antibodies.

For cross-reactivity ELISAs, trimeric recombinant full-length HA proteins were utilized. The HA panel included NC99 (H1) (BEI Resources, NR-48873), Ca09 (H1) (BEI Resources, NR-42635), A/Singapore/1/1957 (H2) (BEI Resources, NR-52249), A/Wisconsin/67/2005 (H3) (BEI Resources, NR-49237), A/Vietnam/1204/2004 (H5) (VRC, NIH), A/Anhui/1/2013 (H7) (BEI Resources, NR-44365), and A/Hong Kong/33982/2009 (H9) (BEI Resources, NR-41792). Recombinant HA protein (100 ng/well) diluted in sodium carbonate/bicarbonate coating buffer (pH 9.5) was added to the wells of 96-half-well ELISA microplates (Greiner Bio-One) and incubated overnight at 4°C. After coating, plates were blocked for 1 hour with 1× casein blocking buffer (prepared from 10× Casein Blocking Buffer; Sigma-Aldrich) supplemented with 2% goat serum (Lampire Biologicals), followed by washing with PBS containing 0.1% Tween 20. Plasma samples were serially diluted in 1× blocking buffer. Wells without antigen were used as negative controls. To detect bound Abs, HRP-conjugated anti–monkey IgG (Fitzgerald, 43C-CB1603) was applied at a 1:5,000 dilution. Plates were developed using 25 μL of 3,3′,5,5′-tetramethylbenzidine (TMB) substrate (Sigma-Aldrich), and the reaction was stopped after 30 minutes with 25 μL of 2N H_2_SO_4_. Absorbance was measured at 450 nm using an Elx800 Absorbance Microplate Reader (BioTek). For each plasma dilution, the optical density (OD) of non–HA-coated wells was subtracted from that of HA-coated wells. The threshold titer was defined as the dilution at which the OD value reached at least 3 times the background signal, measured in wells containing only 1× blocking buffer and stabilized stem protein (without sample).

### Statistics.

Several analytical methods were employed to test the hypotheses in this study. To compare HA head and HA stem responses across different vaccine groups, a 2-way ANOVA was conducted, followed by Fisher’s least significant difference (LSD) test for group comparisons. For cross-reactivity analyses across multiple time points, 1-way ANOVA models were applied, with pairwise comparisons performed using Tukey’s post hoc analysis. When comparing 2 vaccine groups, a 2-tailed Mann-Whitney test was used. For all models, statistical significance was defined as a *P* value of 0.05 or less. Analysis was performed using Prism 10.1.2 (GraphPad).

### Study approval.

The animal care and use protocol was adherent to the US Animal Welfare Act and Regulations. Permission was granted to perform all animal experiments by the Wake Forest University Institutional Animal Care and Use Committee. AGMs were housed and cared for in accordance with state, federal, and institute policies in facilities accredited by the American Association for Accreditation of Laboratory Animal Care (AAALAC) under standards established in the Animal Welfare Act and the NIH *Guide for the Care and Use of Laboratory Animals* (National Academies Press, 2011).

### Data availability.

All data in the manuscript are provided in the associated Supporting Data Values file.

## Author contributions

KFC and MAAM were responsible for manuscript conceptualization. KFC, BCH, and CLP developed methodology and performed the investigation. MS produced reagents. KFC and MAAM were responsible for visualization of the data. MAAM obtained funding. MAAM and MK were responsible for project administration and supervision. KFC and MAAM wrote the first draft of the manuscript. All authors contributed to manuscript review and editing.

## Funding support

This work is the result of NIH funding, in whole or in part, and is subject to the NIH Public Access Policy. Through acceptance of this federal funding, the NIH has been given a right to make the work publicly available in PubMed Central.

- National Institute of Allergy and Infectious Diseases, NIH grants 5R01AI146059 and T32AI007401 (to MAAM).
- National Cancer Institute Cancer Center Support Grant P30CA012197 (to R. Mesa, PI).
- NIH grant P40 OD010965 (partial funding for the The Vervet Research Colony; M. Jorgensen, PI).
- Vaccine Research Center, National Institute of Allergy and Infectious Diseases, NIH (to MK).

## Supplementary Material

Supplemental data

Supporting data values

## Version 1 11/10/2025

Electronic publication
