# Supplementary material for: H1N1 challenge results in rapid recall of stem-specific immunity in HA stem nanoparticle–vaccinated newborn monkeys
Source: JCI Insight. 2025 Nov 10;10(21):e194932. doi: 10.1172/jci.insight.194932 (PMC12643491; doi:10.1172/jci.insight.194932)
Supplement: Supplemental data [file jciinsight-10-194932-s137.pdf]

Supplementary Table 1. Viral load (viral genomes/ml) in the BAL from vaccinated and control newborns d7 following challenge.

H1ssF+AddaVax vg/ml (animal #)	Ctrl vg/ml (animal #)
1,560,000 (2651)	544,000 (2644)
727,000 (2656)	1,450,000 (2664)
1,760,000 (2657)	6,230,000 (2808)
1,720,000 (2659)	2,344,603 (2737)
18,400 (2661)	131,000,000 (2785)
116,004 (2698)	3,367,954 (2720)
451,895 (2717)	5,996,412 (2723)
448,912 (2722)	1,456,797 (2729)
	4,700,452 (2741)

Data were previously published in Crofts et al. 2025. Nat Commun.16:3785 and are shown here in alignment with author reuse policies of the publisher.

Supplementary Table 2. Newborn AGM enrollment data and identifiers.

Animal I.D	Vaccine Received	Sex	Age 1st dose (days)	Symbol
2644	PBS	M	3	▽
2664	PBS	F	3	△
2808	PBS	M	4	◇
2737	mRNA Luciferase-LNP	M	5	○
2785	mRNA Luciferase-LNP	F	4	□
2720	H1ssF	M	3	● (blue)
2723	H1ssF	M	4	■ (blue)
2729	H1ssF	F	5	▲ (blue)
2741	H1ssF	F	3	◆ (blue)
2651	H1ssF+AddaVax	F	5	● (red)
2656	H1ssF+AddaVax	M	3	■ (red)
2657	H1ssF+AddaVax	F	3	▲ (red)
2659	H1ssF+AddaVax	M	4	◆ (red)
2661	H1ssF+AddaVax	F	5	○ (red)
2698	H1ssF+AddaVax	M	5	□ (red)
2717	H1ssF+AddaVax	M	5	△ (red)
2722	H1ssF+AddaVax	F	4	◇ (red)

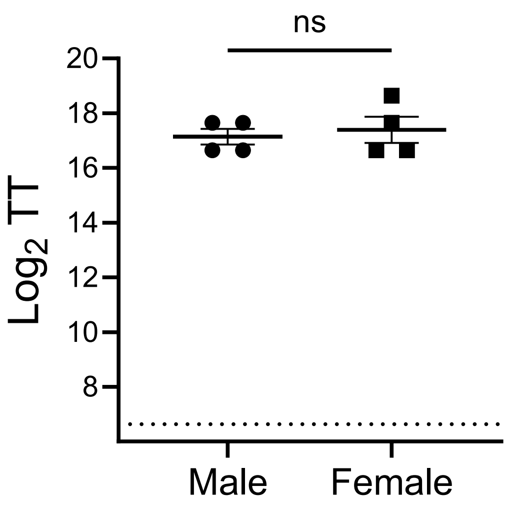

Supplemental Figure 1. Male and female newborns vaccinated with H1ssF+AddaVax have similar stem-specific IgG levels at d41/45p.b.

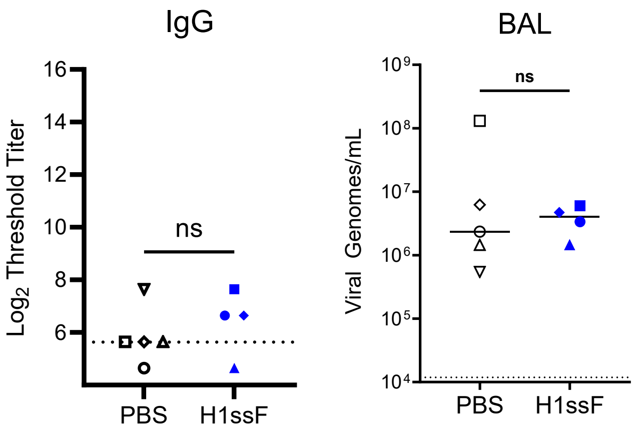

Supplemental Figure 2. Newborns administered PBS or non-adjuvanted H1ssF have similar stem-specific IgG at d41/45p.b and viral titers at d7p.c. Data were previously published in Crofts et al. 2025. Nat Commun.16:3785 and are shown here in alignment with author reuse policies of the publisher.

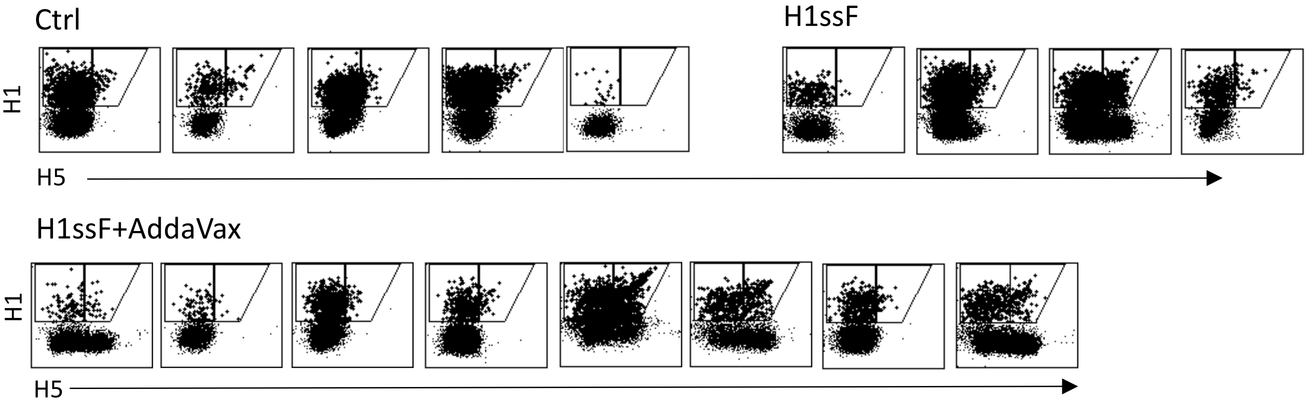

Supplemental Figure 3. HA head and stem-specific B cells in newborns on d7p.c. Samples were gated as in 1B prior to evaluation of H1+H5- and H1+H5+ populations.

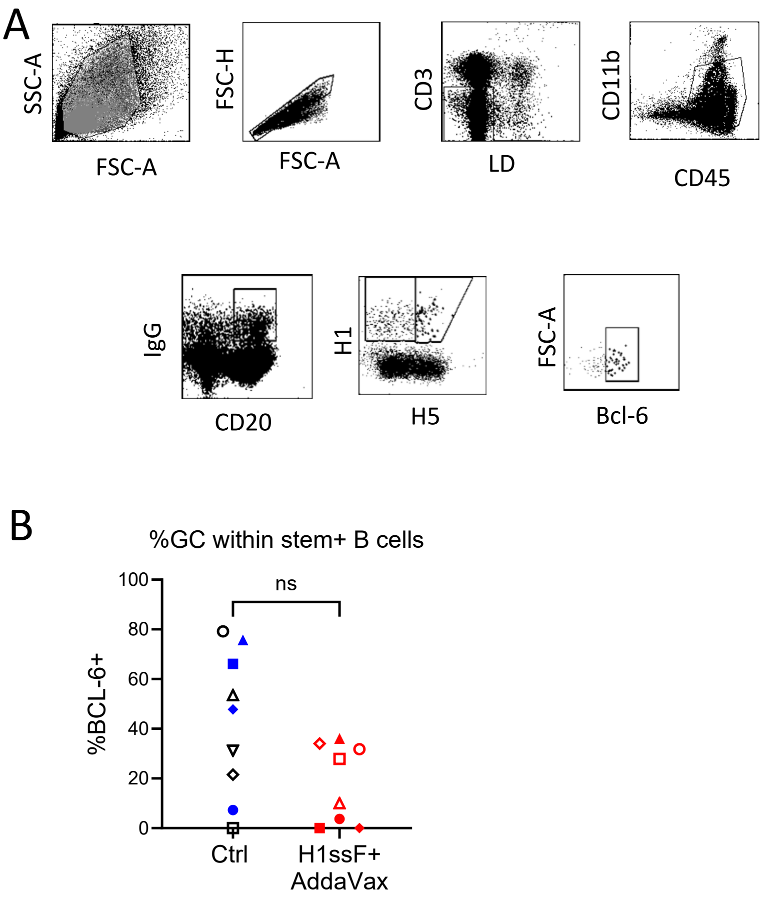

Supplemental Figure 4. Vaccination with H1ssF +AddaVax does not promote significant changes in the proportion of GC B cells in the HA stem+CD20+IgG+ population following challenge. Gating strategy to identify GC B cells (%BCL-6+) within the HA stem+IgG+ population (A). Percentage of GC B cells within the HA stem+ CD20+IgG+ population (B). Ctrl (PBS/Luc mRNA black symbols and H1ssF blue symbols, n=9), H1ssF+AddaVax (red symbols, n=8). The first five panels in (A) are the same as in Figure 1B. Statistical significance was determined using a two-tailed Mann-Whitney test. Not significant p>0.05 (ns). LD, live/dead viability stain.

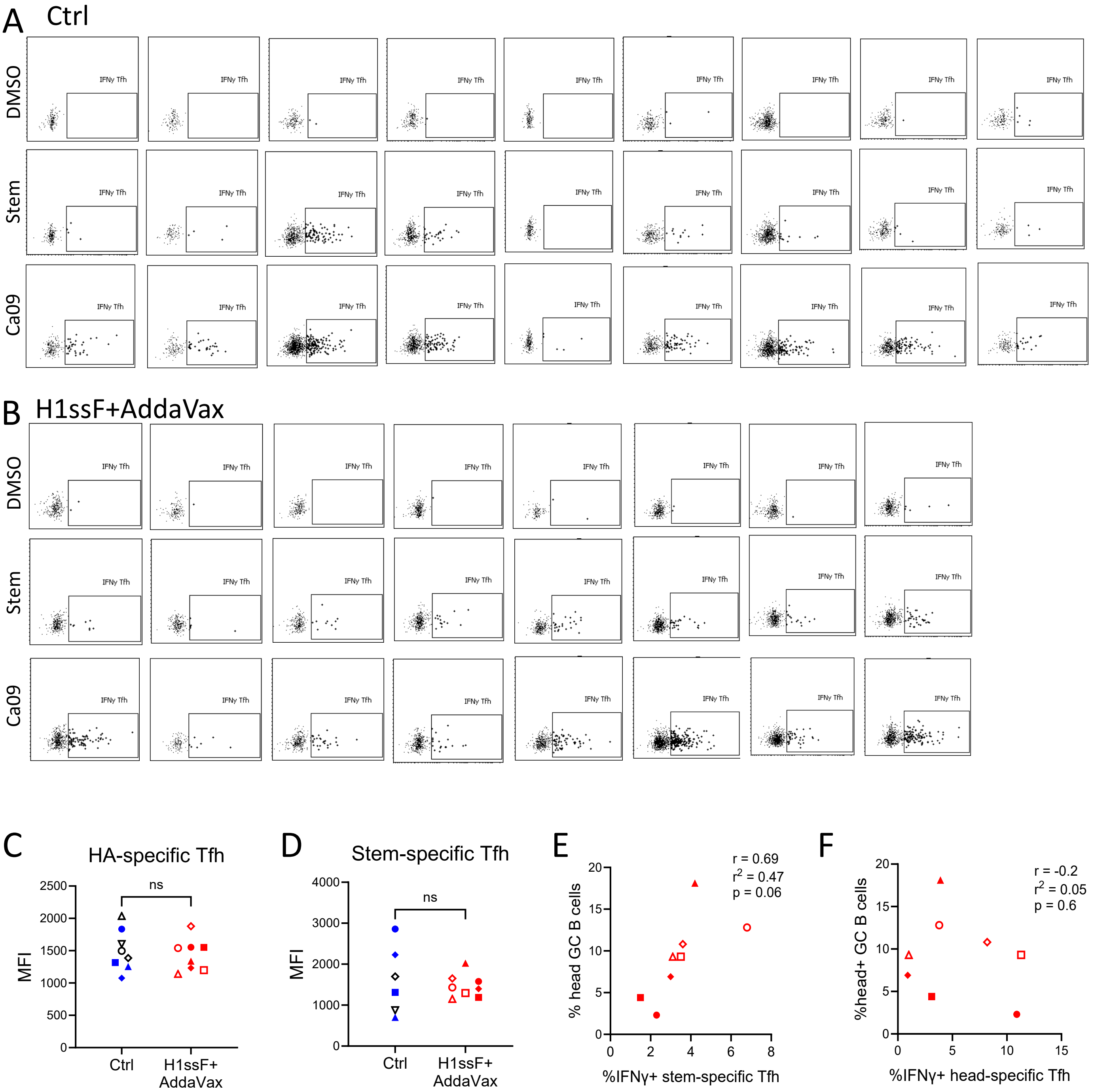

Supplemental Figure 5. Vaccination with H1ssF+AddaVax does not promote differences in the level of IFNγ expression in Tfh. Flow plots gated on IFNγ producing cells within the Tfh (CD3+CD4+PD-1hiBCL-6+FoxP3-) population for Ctrl (A) and H1ssF+AddaVax (B) animals. Geometric mean fluorescent intensity (MFI) of the IFNγ+ cells within the Tfh population following stimulation with HA-Ca09 (C) or NC99-stem (D) peptide pools. Pearson's correlation analysis was performed using the percentage of HA head+ GC B cells and the percentage of IFNγ+ stem-specific Tfh cells in the TBLN at d7 p.c. in H1ssF+AddaVax animals (E). Pearson's correlation was performed using the percentage of HA head+ GC B cells and the percentage of IFNγ+ head-specific Tfh cells in the TBLN d7 p.c. in H1ssF+AddaVax animals (F). Ctrl (PBS/Luc mRNA black symbols and H1ssF blue symbols, n=9), H1ssF+AddaVax (red symbols, n=8). Statistical significance was determined using a two-tailed Mann-Whitney test. Not significant p>0.05 (ns).

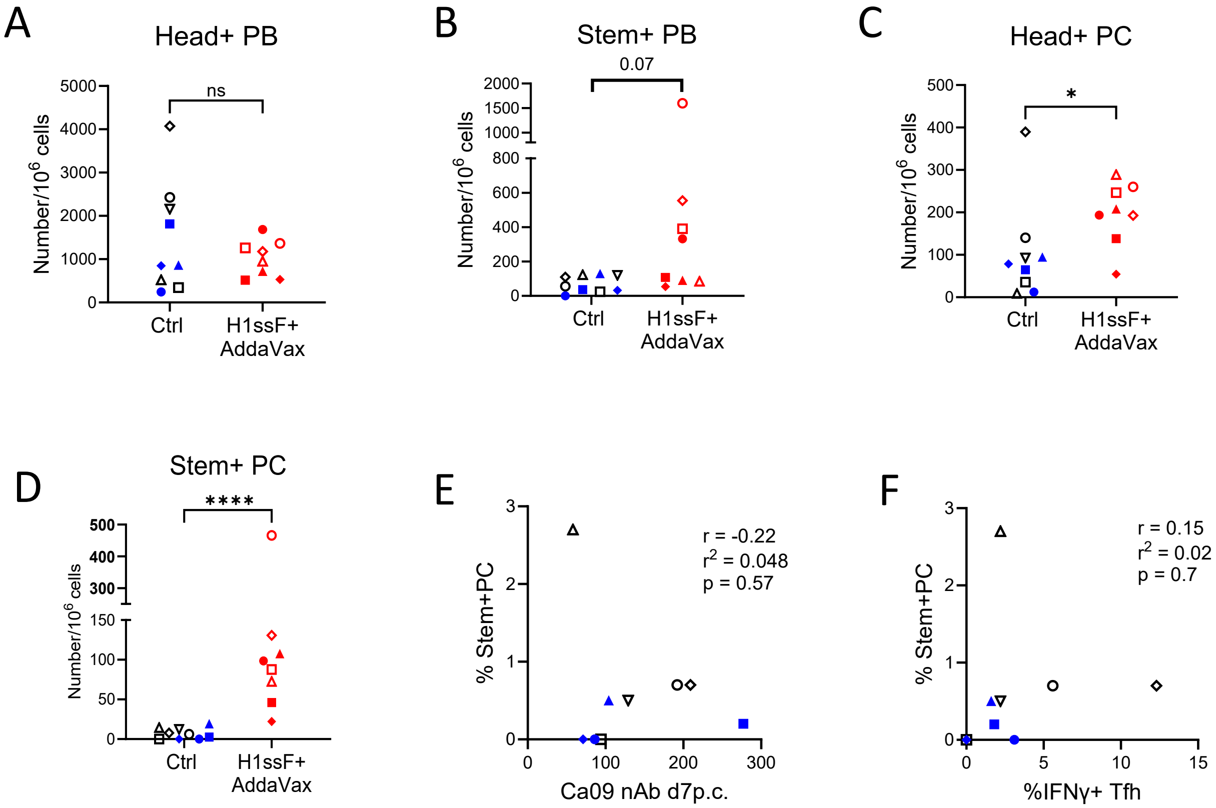

Supplemental Figure 6. Vaccination with H1ssF+AddaVax promotes increases in the number of HA-specific PCs in the TBLN after challenge. The number of HA head+ (A) and HA stem+ PBs (B) in the TBLN d7p.c. The number of HA head+ (C) and HA stem+ (D) PCs in the TBLN d7p.c. Pearson's correlation was performed using the percentage of HA stem+ PCs in the TBLN p.c. against the Ca09 nAb in the plasma at d7p.c. in Ctrl animals (E). Pearson's correlation was performed on percentage of HA stem+ plasma cells in the TBLN at d7 p.c. against the percentage of IFNγ+stem+ Tfh cells in the TBLN d7p.c. in Ctrl animals (F) Ctrl (PBS/Luc mRNA black symbols and H1ssF blue symbols, n=9), H1ssF+AddaVax (red symbols, n=8). Statistical significance was determined using a two-tailed Mann-Whitney test (A, B). Not significant $p>0.05$ (ns), *$p< 0.05$, ****$p\leq 0.0001$. nAb titers were previously published in Crofts et al. 2025. Nat Commun.16:3785 and are shown here in alignment with author reuse policies of the publisher.

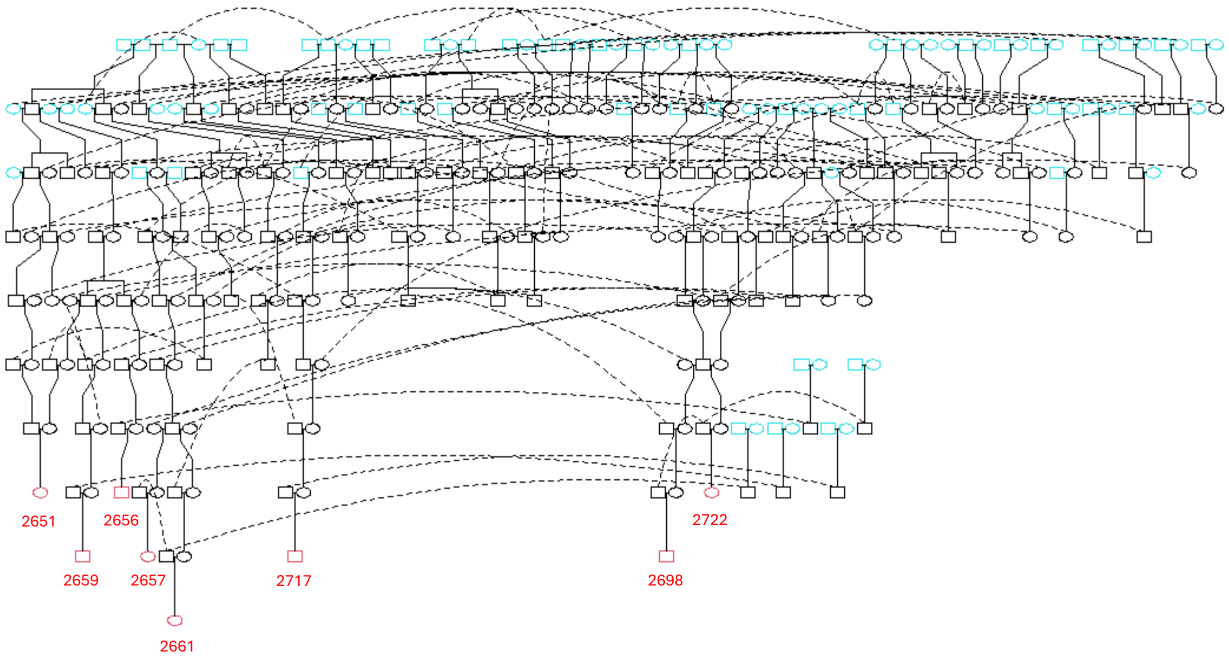

	2651	2656	2657	2659	2661	2698	2717	2722
2651	0.508301	0.009155	0.004395	0.034729	0.004089	0.013245	0.009918	0.018799
2656	0.009155	0.5	0.001923	0.004822	0.002518	0.003998	0.002365	0.006775
2657	0.004395	0.001923	0.5	0.001297	0.150742	0.002365	0.001472	0.00296
2659	0.034729	0.004822	0.001297	0.5	0.001671	0.004532	0.005013	0.00473
2661	0.004089	0.002518	0.150742	0.001671	0.5	0.002159	0.001209	0.002213
2698	0.013245	0.003998	0.002365	0.004532	0.002159	0.5	0.002823	0.147461
2717	0.009918	0.002365	0.001472	0.005013	0.001209	0.002823	0.5	0.003082
2722	0.018799	0.006775	0.00296	0.00473	0.002213	0.147461	0.003082	0.5

Supplemental Figure 7. Pedigree of newborns used in study. Newborns in the study are represented in the pedigree by red symbols. Blue symbols indicate founder animals, either original to the colony at WFUSM or introduced into the colony in 2018 to increase diversity. The bottom table shows the kinship matrix.
